# Prognostic Value of Neutrophil-to-Lymphocyte Ratio in Locally Advanced Rectal Cancer Treated with Neoadjuvant Concurrent Chemoradiotherapy and Robotic-Assisted Resection

**DOI:** 10.32604/or.2025.069397

**Published:** 2026-02-24

**Authors:** Yen-Cheng Chen, Tsung-Kun Chang, Wei-Chih Su, Yung-Sung Yeh, Po-Jung Chen, Tzu-Chieh Yin, Ching-Chun Li, Ching-Wen Huang, Hsiang-Lin Tsai, Jaw-Yuan Wang

**Affiliations:** 1 Graduate Institute of Clinical Medicine, College of Medicine; Kaohsiung Medical University, Kaohsiung, 80708, Taiwan; 2 Division of Colorectal Surgery, Department of Surgery, Kaohsiung Medical University Hospital, Kaohsiung Medical University, Kaohsiung, 80708, Taiwan; 3 Department of Surgery, Faculty of Medicine, College of Medicine, Kaohsiung Medical University, Kaohsiung, 80708, Taiwan; 4 Department of Surgery, Faculty of Post-Baccalaureate Medicine, College of Medicine, Kaohsiung Medical University, Kaohsiung, 80708, Taiwan; 5 Division of Trauma and Surgical Critical Care, Department of Surgery, Kaohsiung Medical University Hospital, Kaohsiung Medical University, Kaohsiung, 80708, Taiwan; 6 Department of Emergency Medicine, Faculty of Post-Baccalaureate Medicine, College of Medicine, Kaohsiung Medical University, Kaohsiung, 80708, Taiwan; 7 Graduate Institute of Injury Prevention and Control, College of Public Health, Taipei Medical University, Taipei, 11031, Taiwan; 8 Division of Colorectal Surgery, Department of Surgery, Kaohsiung Medical University Gangshan Hospital, Kaohsiung, 820, Taiwan; 9 Department of Surgery, Division of Colorectal Surgery, Kaohsiung Municipal Hsiaokang Hospital, Kaohsiung, 812, Taiwan; 10 Graduate Institute of Medicine, College of Medicine, Kaohsiung Medical University, Kaohsiung, 80708, Taiwan; 11 Center for Cancer Research, Kaohsiung Medical University, Kaohsiung, 80708, Taiwan; 12 Center for Disease Multi-omincs Research, Kaohsiung Medical University, Kaohsiung, 80708, Taiwan

**Keywords:** Locally advanced rectal cancer, neoadjuvant concurrent chemoradiotherapy, robotic-assisted surgery, neutrophil-to-lymphocyte ratio, carcinoembryonic antigen

## Abstract

**Background:**

The long-term outcomes of robotic-assisted surgery and the prognostic significance of the pretreatment neutrophil-to-lymphocyte ratio (NLR) in locally advanced rectal cancer (LARC) remain uncertain. This study aimed to assess the long-term outcomes of patients with LARC undergoing robotic-assisted surgery and to determine the prognostic value of pretreatment NLR.

**Methods:**

We retrospectively reviewed 252 patients with LARC who were treated at a single medical center in Taiwan between January 2012 and January 2023. All patients underwent neoadjuvant concurrent chemoradiotherapy (CRT) followed by robotic-assisted surgery with total mesorectal excision (TME). Patients were stratified into four groups on the basis of pretreatment NLRs and carcinoembryonic antigen (CEA) levels. Univariate and multivariate analyses were conducted to identify prognostic indicators for overall survival (OS) and disease-free survival (DFS).

**Results:**

Patients with a pretreatment NLR of ≥3.2 exhibited significantly worse OS and DFS compared with those with an NLR of <3.2 (OS: 94.4 vs. 116.5 months, *p* = 0.001; DFS: 78.8 vs. 101.7 months, *p* = 0.003). Group A exhibited the poorest prognosis, whereas Group D had the most favorable outcomes. Multivariate analysis revealed NLR ≥ 3.2 as an independent predictor of poor OS (hazard ratio [HR] = 2.306, 95% CI: 1.149–3.747; *p* = 0.001) and DFS (HR = 2.055, 95% CI: 1.341–3.148; *p* = 0.001).

**Conclusion:**

Neoadjuvant concurrent CRT followed by robotic-assisted TME is an effective treatment strategy for LARC. A higher pretreatment NLR (≥3.2) independently predicted worse OS and DFS. Stratification using the NLR in combination with CEA levels may enhance prognostic accuracy for patients undergoing robotic-assisted surgery for LARC.

## 1 Background

Neoadjuvant concurrent chemoradiotherapy (CRT) is widely used in the treatment of locally advanced rectal cancer (LARC) [1–3]. Surgical resection of LARC remains technically demanding because of the narrow pelvic space and complex anatomy [4–6]. Furthermore, local recurrence rates following surgery can reach 15%–31% [6,7]. In consideration of these challenges, neoadjuvant radiotherapy was introduced as a means of reducing tumor volume and improving local recurrence control [5,8]. When combined with total mesorectal excision (TME), the local recurrence rate associated with neoadjuvant radiotherapy can be significantly reduced to 6%–7% [9,10]. Advances in surgical technology have further improved outcomes; robotic systems have been developed that offer high-definition, three-dimensional magnified visualization, along with multidirectional instruments capable of rotation at various angles. These features enable more precise resections and have been associated with improved oncologic outcomes in rectal cancer surgery [5,11,12].

Although the aforementioned developments have improved the local recurrence rates associated with radiotherapy, the effects of such therapy on overall survival (OS) in rectal cancer remain a topic of debate [6]. A randomized controlled trial (RCT) in Germany involving 421 patients with T3 or T4 tumors or node-positive LARC revealed that preoperative CRT improved local control but had no advantage over postoperative CRT in terms of OS [13]. Additionally, a multicenter Dutch RCT that followed up 1861 patients with resectable rectal cancer for 12 years reported a significantly lower local recurrence rate in the preoperative radiotherapy group compared with that in the surgery alone group (5% vs. 11%, *p* < 0.0001); notably, no difference in OS was noted between the two groups [14]. The PROSPECT Trial (Alliance N1048), which compared neoadjuvant CRT with neoadjuvant chemotherapy alone in LARC, demonstrated comparable 5-year disease-free survival (DFS) and OS between the groups, with no clear OS benefit being associated with CRT [15].

Prognostic factors in rectal cancer have long been a subject of clinical interest. Variables such as race, sex, age, pathologic T and N stage, tumor grade, pretreatment and peritreatment carcinoembryonic antigen (CEA) levels, and postoperative chemotherapy have been identified as independent prognostic indicators [16,17]. Achieving pathological complete response (pCR) after CRT, observed in 15%–31% of LARC cases, is also associated with better survival [1,18,19]. Recently, the neutrophil-to-lymphocyte ratio (NLR), an indicator of systemic inflammation, has emerged as another potential prognostic biomarker [20,21]. Our previous study demonstrated that an NLR of ≥3.2 is an independent predictor of poor OS and DFS in patients with LARC who received neoadjuvant CRT and achieved pCR [9].

In consideration of the aforementioned findings, we investigated effective prognostic markers for OS in LARC and elucidated the role of neoadjuvant CRT in LARC. We retrospectively reviewed 252 patients with LARC who underwent neoadjuvant concurrent CRT followed by robotic low anterior resection and TME. Real-world data, including patient characteristics, laboratory results, pathological findings, and long-term survival outcomes, were analyzed.

## 2 Materials and Methods

### 2.1 Patients

#### 2.1.1 Patient Selection

We retrospectively reviewed 695 patients who underwent robotic colorectal cancer resection at Kaohsiung Medical University Hospital, a tertiary academic medical center, between January 2012 and January 2023. Patients with colon cancer, stage IV disease, non-adenocarcinoma histology, or incomplete clinical information were excluded. Ultimately, 252 patients with LARC who received neoadjuvant concurrent CRT followed by robotic low anterior resection were included in the study (Supplementary Fig. S1). Clinical data were obtained from medical records, with these data including patient demographics, routine laboratory results, serum CEA levels, pathological findings, local recurrence, distant metastasis, and survival status. The study protocol was approved by the Institutional Review Board of Kaohsiung Medical University Hospital (KMUHIRB-E(II)-20250271).

#### 2.1.2 Patient Care

The diagnosis of adenocarcinoma in patients with LARC was confirmed through histopathologic examination. Treatment strategies—including surgical resection, chemotherapy, and chemoradiotherapy—were determined through multidisciplinary team cancer conferences, following institutional clinical practice guidelines [8]. LARC was defined on the basis of the following computed tomography (CT) criteria: (1) T3 tumors with >5 mm extramural extension, (2) clinical T4-stage tumors, or (3) CT-detected regional lymph node metastases. The neoadjuvant chemotherapy regimen comprised biweekly FOLFOX (folinic acid, 5-fluorouracil, and oxaliplatin) administered concurrently with long-course radiotherapy (total dose of 5000 cGy delivered in 25 fractions). Following completion of radiotherapy, chemotherapy was continued biweekly until 2–3 weeks prior to surgery. Robotic surgery was performed using the single-docking technique, as described in our previous study [5]. The other aspects of chemotherapy dosing followed protocols established through our institutional experience with colorectal cancer treatment [5,8].

After surgery, the adjuvant chemotherapy with FOLFOX was administered every 2 weeks for a total of 12 cycles. Patients who achieved pCR received fluoropyrimidine-based chemotherapy for up to 6 months, with close follow-up [8]. For those who developed local recurrence or distant metastasis, treatment was guided by current consensus recommendations, including the use of chemotherapy or targeted therapy [22,23].

Patients were stratified according to pretreatment CEA levels (≥5 ng/mL vs. <5 ng/mL) for evaluation of survival outcomes [8]. The pretreatment NLR was also categorized (≥3.2 vs. <3.2) because this threshold was previously identified as a poor prognostic factor in our earlier studies [9]. To further assess the combined prognostic impact of CEA and the NLR, patients were classified into four groups: Group A: NLR ≥ 3.2, CEA ≥ 5 ng/mL; Group B: NLR ≥ 3.2, CEA < 5 ng/mL; Group C: NLR < 3.2, CEA ≥ 5 ng/mL; and Group D: NLR < 3.2, CEA < 5 ng/mL.

### 2.2 Statistical Analysis

Descriptive statistics, including medians, means, and proportions, were used to summarize patient demographics and gene alteration profiles. The end of the follow-up period was defined as the date of death, the last follow-up visit, or 31 December 2024, whichever occurred first. OS was defined as the interval from the date of LARC diagnosis to death from any cause, last follow-up, or the study endpoint. DFS was defined as the time from treatment initiation to the occurrence of local recurrence or distant metastasis or to the date of the last follow-up.

DFS and OS were evaluated using the Kaplan–Meier method, and survival curves were compared using the log-rank test. Univariate and multivariate analyses were performed using the Cox proportional-hazards regression model to identify prognostic factors for OS and DFS. Multicollinearity among variables was assessed using the variance inflation factor (VIF), and variables with a VIF <2 were considered acceptable. The proportional hazards assumption for the Cox regression model was verified using Schoenfeld residuals, and no violations were observed. A two-sided *p* value of <0.05 was considered significant. All statistical analyses were performed using SPSS software, version 20.0 (IBM Corp., Armonk, NY, USA), consistent with our previous studies [8,23].

## 3 Results

### 3.1 Patient Characteristics

Among the 252 patients with LARC, the median age was 63 years, and 63.9% were male. Most patients had American Society of Anesthesiologists (ASA) scores of 2–3, and 93.7% had adequate preoperative nutritional status (albumin >3.5 g/dL). Clinically, 76.2% had T3 disease, 54.4% had N1, and 25.8% had N2 stage tumors.

After surgery, a free circumferential resection margin was achieved in 97.6% of patients. pCR (ypT0N0) was observed in 27.4% of cases, whereas 25.8% and 36.5% had ypT2 and ypT3 disease, respectively. Most tumors were moderately differentiated adenocarcinomas (86.1%), with vascular and perineural invasion observed in 9.9% and 15.5% of patients, respectively.

Before treatment, elevated CEA levels (≥5 ng/mL) were found in 41.3% of patients, and a pretreatment NLR ≥3.2 in 36.1%. During follow-up, local recurrence and distant metastasis occurred in 3.2% and 10.3% of patients, respectively. Detailed clinicopathologic characteristics are summarized in Table 1.

**Table 1:** Summary and characteristics of patients (patients, N = 252)

Characteristic	n (%)
**Age (years, median) (range)**	63 (28–93)
**Sex**	
Male	161 (63.9%)
Female	91 (36.1%)
**BMI kg/m**^**2**^ **(mean) (range)**	24.3 (17.6–41.1)
**ASA score** ^**a**^	
2	155 (61.5%)
3	97 (38.5%)
**Pre-operative albumin level**	
≥3.5 (g/dL)	236 (93.7%)
<3.5 (g/dL)	16 (6.3%)
**Clinical T stage**	
cT1	2 (0.8%)
cT2	19 (7.5%)
cT3	192 (76.2%)
cT4a	23 (9.1%)
cT4b	16 (6.4%)
**Clinical N stage**	
cN0	50 (19.8%)
cN1	137 (54.4%)
cN2	65 (25.8%)
**Circumferential resection margin**	
Positive	6 (2.4%)
Negative	246 (97.6%)
**Pathologic T stage**	
ypT0	71 (28.2%)
ypT1	20 (7.9%)
ypT2	65 (25.8%)
ypT3	92 (36.5%)
ypT4	4 (1.6%)
**Pathologic N stage**	
ypN0	195 (77.4%)
ypN1	49 (19.4%)
ypN2	8 (3.2%)
**Pathological complete response**	69 (27.4%)
**Histology(adenocarcinoma)** ^**b**^	
Well differentiated	26 (10.3%)
Moderately differentiated	217 (86.1%)
Poorly differentiated	9 (3.6%)
**Vascular invasion**	
Yes	25 (9.9%)
No	227 (90.1%)
**Perineurial invasion**	
Yes	39 (15.5%)
No	213 (84.5%)
**CEA ≥ 5 (ng/mL)**	
Yes	104 (41.3%)
No	148 (58.7%)
**NLR ≥ 3.2**	
Yes	91 (36.1%)
No	161 (63.9%)
**Local recurrence**	
Yes	8 (3.2%)
No	244 (96.8%)
**Distant metastasis**	
Yes	26 (10.3%)
No	226 (89.7%)

Note: ^a^ASA: American Society of Anesthesiologists; ^b^For pathologic complete response patients, histology status derived from diagnostic biopsy specimen; BMI: body mass index; yp: post-therapy pathologic; CEA: carcinoembryonic antigen; NLR: neutrophil lymphocyte ratio.

### 3.2 Survival and Treatment Outcomes

**Figure 1:**
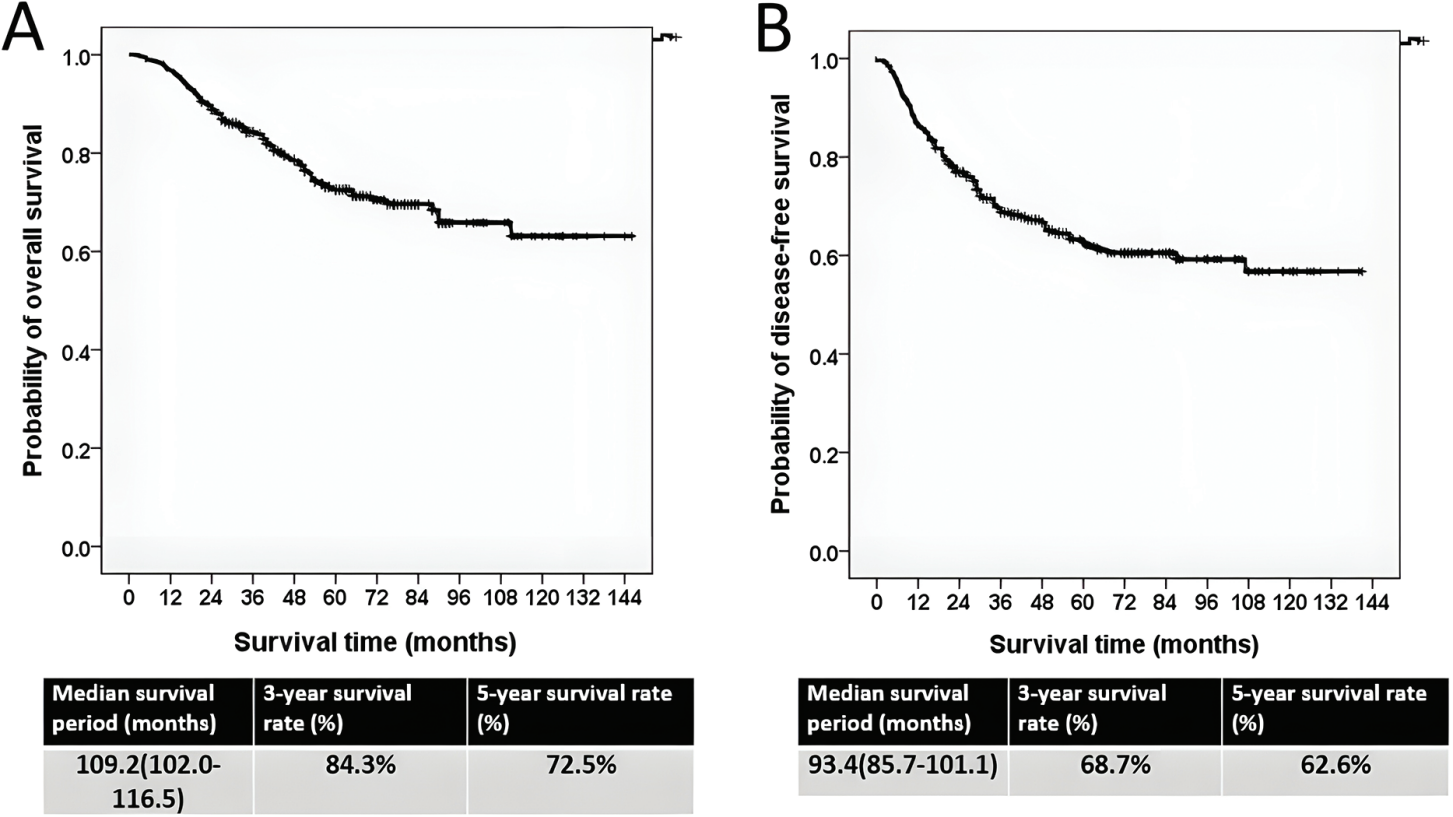
The Kaplan–Meier survival curve. (**A**) Overall survival curve. (**B**) Disease-free survival curve

The median follow-up duration was 61.2 months (range, 7.4–146.7 months). For all patients, the median OS and DFS were 109.2 and 93.4 months, with estimated 5-year OS and DFS rates of 72.5% and 62.6%, respectively (Fig. 1A,B).

**Figure 2:**
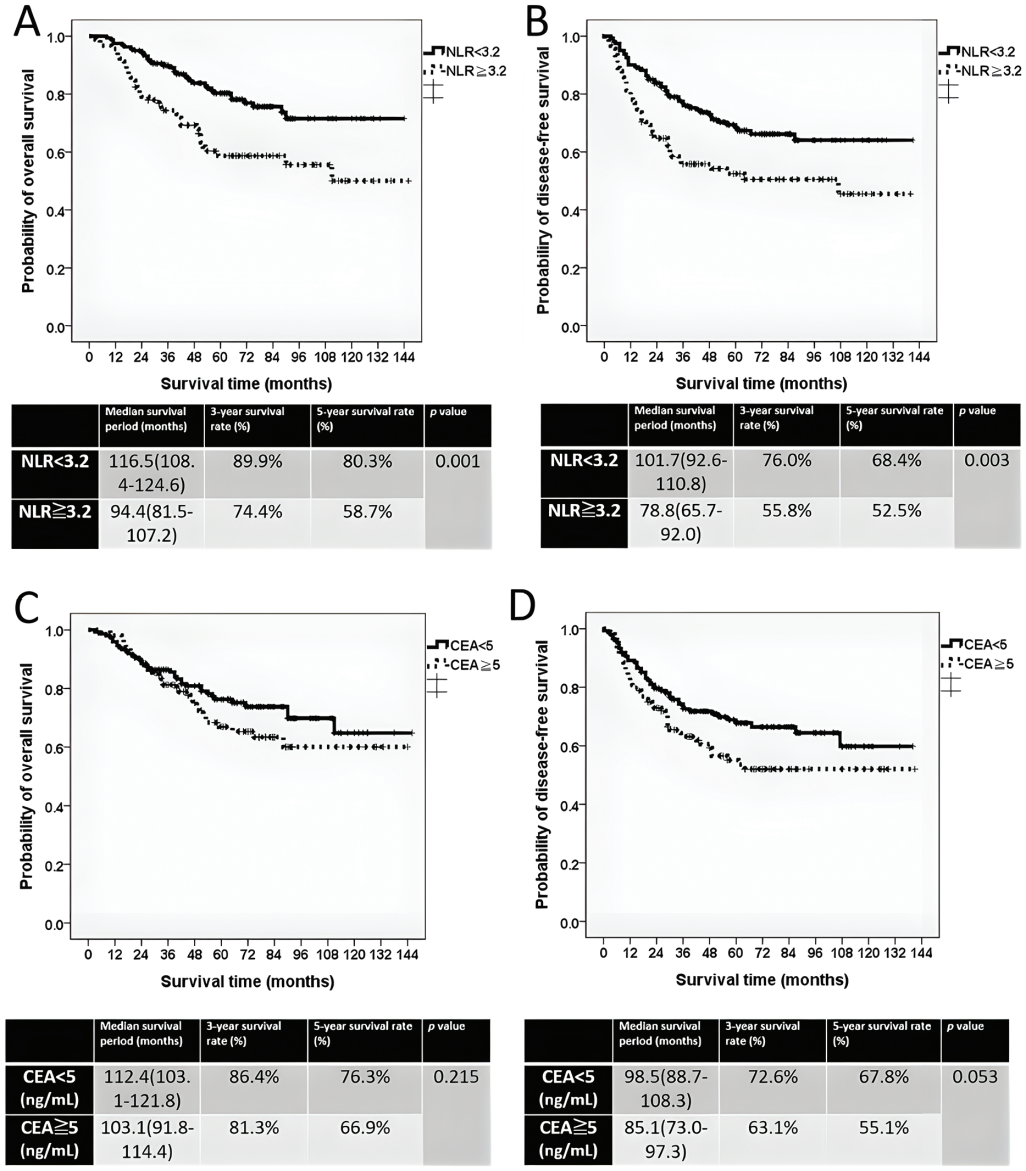
The Kaplan–Meier survival curve of two groups. (**A**) Overall survival curve stratified by pretreatment NLR ≥ 3.2 and NLR < 3.2. (**B**) Disease-free survival curve stratified by pretreatment NLR ≥ 3.2 and NLR < 3.2. (**C**) Overall survival curve stratified by pretreatment CEA ≥ 5 ng/mL and CEA < 5 ng/mL. (**D**) Disease-free survival curve stratified by pretreatment CEA ≥ 5 ng/mL and CEA < 5 ng/mL

The patients were stratified into two groups on the basis of the pretreatment NLR: NLR ≥ 3.2 and NLR < 3.2. Patients with NLR ≥ 3.2 showed significantly poorer survival than those with NLR < 3.2. Median OS was 94.4 vs. 116.5 months (*p* = 0.001), and median DFS was 78.8 vs. 101.7 months (*p* = 0.003) (Fig. 2A,B).

Pretreatment CEA levels (≥5 ng/mL vs. <5 ng/mL) were used to stratify patients for survival analysis. In contrast, CEA levels were not significantly associated with survival. Median OS was 112.4 months for patients with CEA < 5 ng/mL and 103.1 months for those with CEA ≥ 5 ng/mL (*p* = 0.215), whereas median DFS was 98.5 vs. 85.1 months (*p* = 0.053) (Fig. 2C,D).

Patients were divided into four subgroups according to pretreatment NLR and CEA levels:

**Group A:** NLR ≥ 3.2 and CEA ≥ 5 ng/mL (13.9%),

**Group B:** NLR ≥ 3.2 and CEA < 5 ng/mL (22.2%),

**Group C:** NLR < 3.2 and CEA ≥ 5 ng/mL (27.4%), and

**Group D:** NLR < 3.2 and CEA < 5 ng/mL (36.5%).

**Figure 3:**
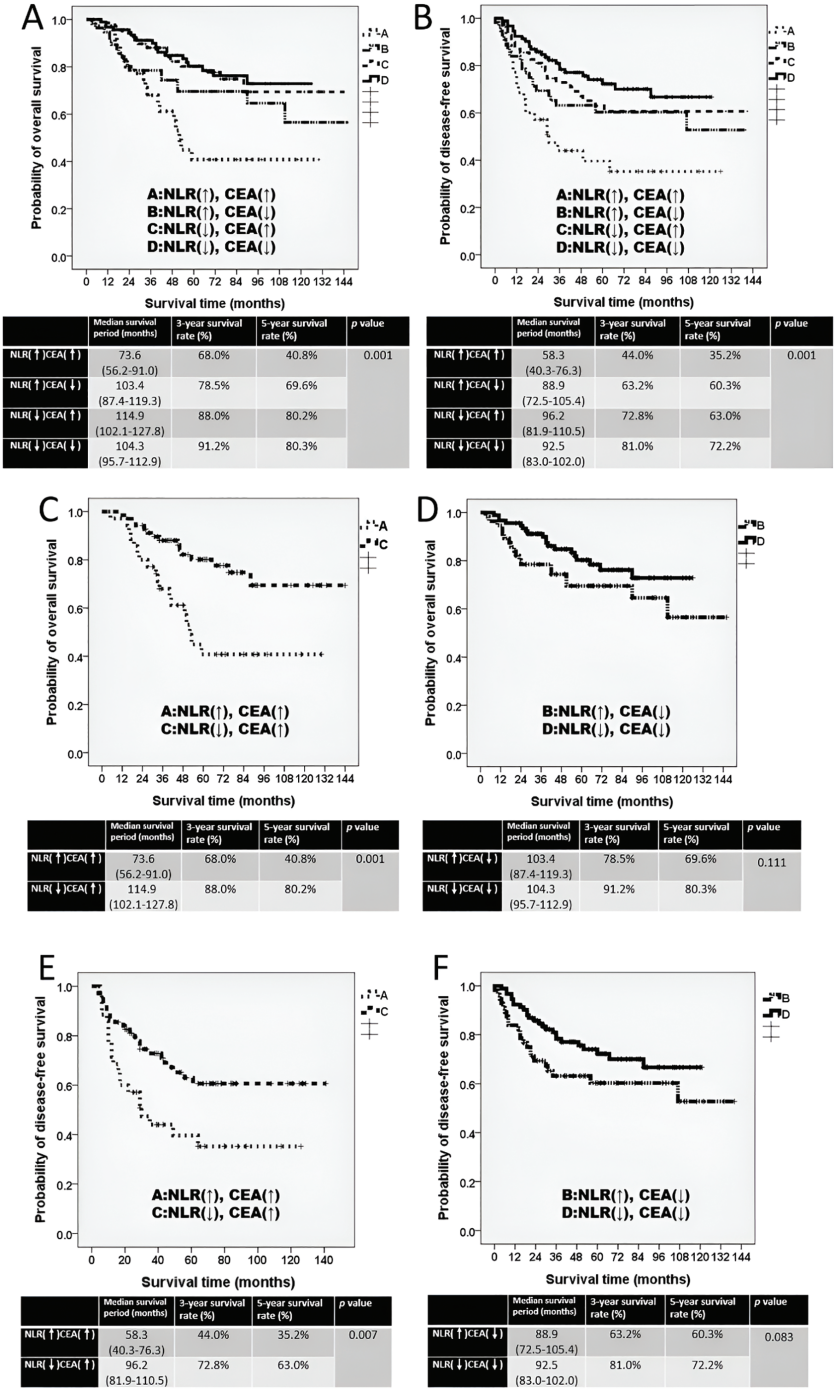
The Kaplan–Meier survival curve of four subgroups. (**A**) Overall survival curve for four subgroups. (**B**) Disease-free survival curve for four subgroups. (**C**) Overall survival curve comparing groups A and C. (**D**) Overall survival curve comparing groups B and D. (**E**) Disease-free survival curve comparing groups A and C. (**F**) Disease-free survival curve comparing groups B and D. The four subgroups were defined as follows: Group A, NLR ≥ 3.2 and CEA ≥ 5 ng/mL (NLR↑, CEA↑); Group B, NLR ≥ 3.2 and CEA < 5 ng/mL (NLR↑, CEA↓); Group C, NLR < 3.2 and CEA ≥ 5 ng/mL (NLR↓, CEA↑); and Group D, NLR < 3.2 and CEA < 5 ng/mL (NLR↓, CEA↓). ↑ indicates high values (≥ cutoff); ↓ indicates low values (<cutoff)

For OS: Group A demonstrated the poorest survival (median OS, 73.6 months), whereas Group C demonstrated the longest median OS, at 114.9 months. However, Group D exhibited the highest estimated 3-year and 5-year OS rates, which were 91.2% and 80.3%, respectively (Fig. 3A, *p* = 0.001). Among patients with elevated CEA, those with NLR < 3.2 (Group C) had significantly longer OS than those with NLR ≥ 3.2 (Group A) (*p* = 0.001; Fig. 3C). In the CEA < 5 ng/mL subgroup, OS tended to be longer in Group D than in Group B, although the difference was not significant (*p* = 0.111; Fig. 3D).

For DFS: The DFS pattern was consistent with OS findings. Group A showed the shortest DFS (median 58.3 months). Group C exhibited the longest median DFS, at 96.2 months, whereas Group D exhibited the highest estimated 3-year and 5-year DFS rates at 81.0% and 72.2%, respectively (Fig. 3B, *p* = 0.001). Among patients with CEA ≥ 5 ng/mL, those with NLR < 3.2 (Group C) had significantly superior DFS compared with Group A (*p* = 0.007; Fig. 3E). In patients with CEA < 5 ng/mL, DFS was higher in Group D than in Group B, but without statistical significance (*p* = 0.083; Fig. 3F).

### 3.3 Prognostic Factors for Patient Survival

Prognostic factors for OS and DFS were analyzed using Cox regression models (Table 2). On univariable analysis, age ≥ 65 years, pathologic T3–T4 stage, lymph-node metastasis, perineural invasion, and pretreatment NLR ≥ 3.2 were associated with worse OS, whereas pCR predicted better outcomes. In the multivariable model, age ≥ 65 years (HR = 1.820; *p* = 0.018) and NLR ≥ 3.2 (HR = 2.306; *p* = 0.001) remained independent adverse prognostic factors. The NLR and CEA interaction was not significant (*p* = 0.572).

**Table 2:** Results of univariate and multivariate analysis of prognostic indicators for overall survival and disease-free survival

Parameters	Overall survival				Disease-free survival			
	Univariate analysis		Multivariable analysis		Univariate analysis		Multivariable analysis	
	HR^a^ (95% CI^b^)	*p* value	HR^a^ (95% CI^b^)	*p* value	HR^a^ (95% CI^b^)	*p* value	HR^a^ (95% CI^b^)	*p* value
**Age (years)**	1.651 (1.032–2.640)	0.036*	1.820 (1.110–2.982)	0.018*	1.276 (0.847–1.922)	0.244	1.474 (0.963–2.257)	0.074
≥65 vs. <65 (107/145)								
**Sex**	0.939 (0.733–1.202)	0.615	0.918 (0.546–1.541)	0.745	0.953 (0.769–1.181)	0.660	0.971 (0.776–1.214)	0.794
Female vs. Male (91/161)								
**Tumor depth**	1.631 (1.019–2.630)	0.041*	1.122 (0.636–1.983)	0.692	1.694 (1.124–2.552)	0.012*	1.087 (0.670–1.763)	0.736
T3 + T4 vs. T1 + T2 (96/156)								
**LN**^**c**^ **metastasis**	1.677 (1.012–2.781)	0.045*	1.437 (0.777–2.658)	0.247	1.755 (1.130–2.727)	0.012*	1.457 (0.859–2.472)	0.163
N(+) vs. N0 (57/195)								
**pCR** ^**d**^	0.465 (0.249–0.868)	0.016*	0.524 (0.253–1.084)	0.081	0.371 (0.206–0.668)	0.001*	0.432 (0.222–0.843)	0.014*
Yes vs. No (22/175)								
**Histology**	0.974 (0.238–3.979)	0.970	1.144 (0.268–4.879)	0.856	1.037 (0.328–3.278)	0.951	1.277 (0.393–4.145)	0.684
PD vs. MD + WD^**e**^ (9/243)								
**Vascular invasion**	1.244 (0.596–2.600)	0.561	1.010 (0.502–2.031)	0.651	1.347 (0.717–2.532)	0.354	1.304 (0.754–2.255)	0.586
Yes vs. No (25/227)								
**Perineurial invasion**	1.866 (1.053–3.309)	0.033*	1.832 (0.975–3.443)	0.060	1.781 (1.074–2.953)	0.025*	1.568 (0.899–2.735)	0. 113
Yes vs. No (38/214)								
**Pre-Tx NLR** ^**f**^	2.150 (1.345–3.437)	0.001*	2.306 (1.149 –3.747)	0.001*	1.856 (1.232–2.798)	0.003*	2.055 (1.341–3.148)	0.001*
≥3.2/ vs. <3.2 (91/161)								
**Pre-Tx CEA** ^**g**^	1.344 (0.840–2.151)	0.217	1.004 (0.595–1.691)	0.987	1.490 (0.990–2.243)	0.056	1.163 (0.748–1.808)	0.504
≥5 vs. <5 (ng/mL) (104/148)								

Note: ^a^HR: hazard ratio, ^b^CI: confidence interval, ^c^LN: lymph node, ^d^pCR: pathologic complete response, ^e^PD: poorly differentiated, MD: moderately differentiated, WD: well differentiated, ^f^NLR: neutrophil-to-lymphocyte ratio, ^g^CEA: carcinoembryonic antigen. *p < 0.05. The “+” symbol indicates either a combination of categories (e.g., T3 + T4, MD + WD) or a positive status (e.g., N(+)).

Similarly, for DFS, pathologic T3–T4 stage, lymph-node metastasis, perineural invasion, and NLR ≥ 3.2 were linked to poorer outcomes, whereas pCR was protective. Multivariate analysis confirmed NLR ≥ 3.2 (HR = 2.055; *p* = 0.001) as an independent poor prognostic factor and pCR (HR = 0.432; *p* = 0.014) as an independent favorable factor. The NLR and CEA interaction was not significant (*p* = 0.639).

No multicollinearity was detected (all VIF < 2), and proportional-hazards assumptions were satisfied (all *p* > 0.05).

## 4 Discussion

This study presents long-term real-world data on the treatment outcomes of LARC in our institution. The estimated 3-year OS and DFS rates were 84.3% and 68.7%, respectively, which are consistent with our previously published findings [1]. The present study had a longer follow-up period and larger sample size than the previous study. Furthermore, the current study estimated 5-year OS and DFS rates of 72.5% and 62.6%, respectively, outcomes that align with those reported in robotic-assisted rectal cancer surgery research [24]. The similarity in OS and DFS rates between the previous and current research supports the representativeness of the current cohort and suggests that the findings are reflective of real-world clinical practice rather than a selected patient subgroup. Moreover, the current findings of median OS and DFS durations of 109.2 and 93.4 months, respectively, indicate that neoadjuvant concurrent CRT followed by robotic-assisted surgery is an effective treatment strategy for LARC.

This study exclusively analyzed patients with LARC who underwent robotic surgery. In our clinical practice, we have established a standardized treatment protocol involving neoadjuvant concurrent CRT followed by robotic-assisted surgery [1,4]. The smooth and stable motion of the robotic arm enables precise lymph node dissection [12,25]. Although rectal cancer surgery can also be performed through laparoscopy or traditional laparotomy, the current study focused solely on robotic surgery to minimize variability arising from different surgical approaches. Consequently, we are confident that the observed differences in survival outcomes are primarily attributable to patients’ pretreatment condition rather than to the surgical technique. Another strength of this study is its extended follow-up duration, with survival outcomes reported over a period exceeding 10 years. Although the retrospective and single-center design of the current study led to it having certain limitations, its use of a homogenous patient cohort and long-term follow-up enhanced the reliability and interpretability of the findings.

Numerous prognostic factors for LARC treatment, such as tumor distance from the anal verge, preoperative and postoperative CEA levels, tumor size and depth, tumor regression grade, lymph node metastasis, distal resection margin, vascular invasion, and perineural invasion, were identified in our previous study [1]. However, in that study, we were unable to establish a simple yet effective prognostic tool for predicting treatment outcomes. In the present study, we proposed the NLR as a potential solution. Research has indicated that NLR values between 2.5 and 5 may predict treatment outcome in patients with LARC [26–28]. For patients with LARC undergoing neoadjuvant CRT followed by radical resection, Colloca et al. reported through a systematic review that an NLR greater than 3 may serve as a reliable prognostic factor [29]. Similarly, our previous study of 478 patients with LARC identified NLR ≥ 3.2 as an independent adverse prognostic factor based on receiver operating characteristic (ROC) analysis [9]. Consistent with these findings, the current study demonstrated that patients with NLR < 3.2 exhibited significantly better OS and DFS compared with those of patients with NLR ≥ 3.2 (Fig. 2A,B). Multivariate analysis confirmed that an NLR of ≥3.2 is an independent predictor of poor survival. Notably, the prognostic value of NLR was not affected by CEA levels.

The NLR is a widely recognized biomarker for predicting cancer prognosis [30,31]. However, the underlying mechanism linking an elevated NLR to poor outcomes in LARC remains unclear [9]. The most commonly proposed explanation involves the interplay between inflammation and immune response [32,33]. Neutrophils reflect systemic inflammation, which can promote tumor cell proliferation and angiogenesis, thereby facilitating cancer progression and metastasis [33]. Conversely, lymphocytes represent the host’s antitumor immune response within the tumor microenvironment; a reduced peripheral lymphocyte count may indicate compromised immune surveillance, contributing to unfavorable treatment outcomes [34]. High NLR reflects a state of systemic inflammation and immune dysregulation. Neutrophils can secrete various pro-tumorigenic cytokines, such as interleukin-6 and vascular endothelial growth factor, which promote tumor growth, angiogenesis, and metastasis. Meanwhile, a relative decrease in lymphocyte count indicates impaired immune surveillance, reducing the host’s ability to mount an effective anti-tumor response [30,31,35]. Neutrophil-derived cytokines, particularly interleukin-8, have been implicated in colorectal cancer progression and treatment response. Elevated interleukin-8 promotes epithelial–mesenchymal transition and chemotherapy resistance, and high serum interleukin-8 levels are associated with poor survival in patients with colorectal liver metastases [36,37]. Overall, the NLR is believed to reflect the balance between tumorigenesis and antitumor immunity. A high NLR is associated with poor prognosis in LARC treatment [9]. In the present study, we confirmed our previous finding that pretreatment NLR ≥ 3.2 is a meaningful threshold for predicting LARC treatment outcomes [9].

CEA is a well-established prognostic biomarker in colorectal cancer management [16,17]. However, as demonstrated in our previous study, a pretreatment CEA level of ≥5 ng/mL cannot serve as an independent prognostic factor in every situation [8]. Typically, additional clinical or pathological features are necessary to accurately evaluate treatment outcomes in LARC [9]. Notably, in the present study, patients with CEA < 5 ng/mL exhibited better OS and DFS than did those with CEA ≥ 5 ng/mL, although these differences did not reach statistical significance. Notably, when CEA levels were analyzed in conjunction with the NLR, more nuanced prognostic distinctions emerged. Reports have suggested that combining the NLR and CEA can more effectively predict colorectal cancer outcomes than either alone can [28]. In our analysis, although Group C (NLR < 3.2, CEA ≥ 5 ng/mL) had the longest median OS and DFS, Group D (NLR < 3.2, CEA < 5 ng/mL) exhibited the highest estimated 3-year and 5-year OS and DFS rates. These outcome differences among the four subgroups become increasingly pronounced when the follow-up exceeded 5 years. We also observed that among patients with CEA ≥ 5 ng/mL, the survival differences between group A and C were particularly marked, which aligns with previous findings linking elevated CEA with an increased recurrence risk and poor prognosis [38]. In such high-risk cases, the pretreatment NLR may be a critical factor in assessing treatment outcomes in LARC.

In this study, we exclusively used the pretreatment NLR to predict LARC outcomes rather than assessing the preoperative or postoperative NLR. Although several studies have identified the NLR as a prognostic biomarker in CRC treatment [39–41], most of the studies have focused on metastatic CRC. For example, Nemoto et al. retrospectively analyzed 71 patients with metastatic CRC undergoing first-line chemotherapy and found that a decreased NLR after 3 months was significantly associated with improved OS and PFS [39]. Liu et al. retrospectively analyzed 128 patients with metastatic CRC who received FOLFOX and targeted therapies (bevacizumab or cetuximab) and reported that a decline in the posttreatment NLR was correlated with longer OS and PFS, whereas an increased posttreatment NLR was associated with worse OS and PFS [40]. Notably, these findings are not directly applicable to our cohort, which exclusively comprised patients with LARC without distant metastasis. Although all patients included in this study received 10–12 weeks of chemotherapy before surgery [1], no association was observed between changes in the NLR and survival outcomes. Moreover, the majority of the patients in our study exhibited sustained NLR elevation after neoadjuvant concurrent CRT, likely due to chemotherapy-induced bone marrow suppression and lymphopenia [42]. Additionally, because of the retrospective design of this study, some preoperative and postoperative NLR data were unavailable. To better determine the prognostic utility of the NLR in LARC, future studies with larger sample sizes and more comprehensive laboratory data are warranted.

Radiotherapy is a key component in the treatment of colorectal cancer, particularly for locally advanced tumors [2,8]. In clinical practice, neoadjuvant concurrent CRT often results in substantial tumor shrinkage, which can facilitate tumor resection [1,4]. However, strong evidence has yet to be obtained that supports radiotherapy offering a survival benefit—specifically, prolonged OS—in patients with LARC [13–15]. Although this is understandable for stage IV rectal cancer, where systemic therapy is the primary treatment [43,44], it is paradoxical in stage III LARC, where surgical resection is the main therapeutic approach [2]. It seems contradictory that radiotherapy facilitates surgical resection but fails to improve OS. Our results provide a potential explanation for this: neoadjuvant concurrent CRT can confer survival benefits only in select subgroups of patients with LARC. Specifically, only patients with a pretreatment NLR of <3.2 demonstrate a favorable response to preoperative radiotherapy. Thus, the NLR may primarily reflect the baseline systemic condition of patients with LARC. To validate this, a study must be conducted that uses comparative data from patients who did not receive preoperative radiotherapy. Given the absence of a control group not receiving CRT, our retrospective study design does not allow for the assessment of the predictive value of NLR in relation to treatment benefit. Instead, our findings highlight the significant prognostic value of pretreatment NLR in the management of patients with LARC.

In addition to the NLR, hematological biomarkers such as the platelet-to-lymphocyte ratio (PLR) have been reported to be effective prognostic indicators in rectal cancer [32]. The underlying mechanism of the PLR as a prognostic indicator is similar to that of the NLR. Platelets contribute not only to hemostasis and thrombosis but also to inflammatory processes and immune regulation through cytokine secretion [45], rendering the PLR a valuable biomarker in cancer prognosis [26]. Ergen et al. conducted a retrospective study involving 53 patients with LARC who underwent neoadjuvant CRT and revealed that patients with PLR ≥ 131 had significantly lower OS and DFS rates than did those with PLR < 131. Furthermore, a multivariate analysis in their study revealed that PLR ≥ 131 was an independent poor prognostic factor DFS in LARC [27]. The relevant literature supports the prognostic utility of both the NLR and the PLR in LARC [27,32]. Hence, the PLR can be considered a promising target for future research. Nevertheless, at present, there are no established treatment guidelines for colorectal cancer patients with elevated NLR or PLR. Based on current evidence, these patients may be considered at higher risk for poor outcomes, and closer follow-up with timely intervention upon early signs of disease progression is suggested [46,47].

This study has several notable limitations. First, its retrospective design and limited sample size may affect the generalizability of the findings. In addition, the investigated patient characteristics were primarily pathological features, whereas key preoperative variables such as pelvic magnetic resonance imaging (MRI) image, tumor size, tumor location (e.g., lower vs. mid-rectum), and patients’ comorbidities were not considered. Additionally, perioperative data, including blood loss, operation time, and surgical complications, were not investigated. This missing information may have introduced selection bias, as patients with missing data might have different clinical characteristics or outcomes compared to those with complete data, potentially affecting the precision and generalizability of our findings. Moreover, we focused on the NLR, selecting this ratio on the basis of findings from our previous research [9], and we did not examine other potentially relevant biomarkers, such as the PLR. Furthermore, changes in the NLR throughout the treatment course, such as postoperative values or those following adjuvant chemotherapy, were not analyzed. Although we proposed a hypothesis regarding the differential effect of radiotherapy on LARC outcomes, no direct evidence was presented to support this claim. In our clinical practice, most patients with LARC received neoadjuvant radiotherapy, limiting our ability to compare outcomes in patients treated with surgery alone. Our proposed NLR + CEA model demonstrated promising prognostic value in patients with LARC undergoing neoadjuvant CRT followed by surgery. However, due to the retrospective and single-center nature of our study, external validation was not feasible. Further prospective trials, such as multicenter programs with larger-scale data collection, standardized laboratory testing schedules, and the use of electronic medical record systems, are warranted to better elucidate the prognostic role of NLR in the treatment of LARC.

## 5 Conclusion

This study provides real-world evidence of long-term oncologic outcomes in patients with LARC treated with neoadjuvant CRT followed by robotic-assisted tumor resection. The study findings support the effectiveness of this treatment strategy. A pretreatment NLR of ≥3.2 was identified as a reliable and independent prognostic factor for both OS and DFS. Furthermore, considering the combination of pretreatment NLRs and CEA levels enhanced prognostic stratification. Further research is warranted to elucidate the potential interaction between neoadjuvant CRT and systemic inflammatory markers such as the NLR in the management of LARC.

## Supplementary Materials



## Data Availability

All relevant data and information can be obtained from the corresponding author upon reasonable request.

## Supplementary Materials

The supplementary material is available online at https://www.techscience.com/doi/10.32604/or.2025.069397/s1.

## Abbreviations

ASA: American Society of Anesthesiologists
CEA: Carcinoembryonic antigen
CI: Confidence interval
CRT: Chemoradiotherapy
CT: Computed tomography
DFS: Disease-free survival
FOLFOX: Folinic acid, 5-fluorouracil, and oxaliplatin
HR: Hazard ratio
LARC: Locally advanced rectal cancer
MRI: Magnetic resonance imaging
NLR: Neutrophil-to-lymphocyte ratio
OS: Overall survival
pCR: Pathological complete response
PLR: Platelet-to-lymphocyte ratio
RCT: Randomized controlled trial
ROC: Receiver operating characteristic
TME: Total mesorectal excision
VIF: Variance inflation factor
