# Supplementary material for: Prognostic Value of Neutrophil-to-Lymphocyte Ratio in Locally Advanced Rectal Cancer Treated with Neoadjuvant Concurrent Chemoradiotherapy and Robotic-Assisted Resection
Source: Oncol Res. 2026 Feb 24;34(3):17. doi: 10.32604/or.2025.069397 (PMC12963664; doi:10.32604/or.2025.069397)
Supplement: Supplementary file 1 [file OncolRes-34-69397-s001.docx]

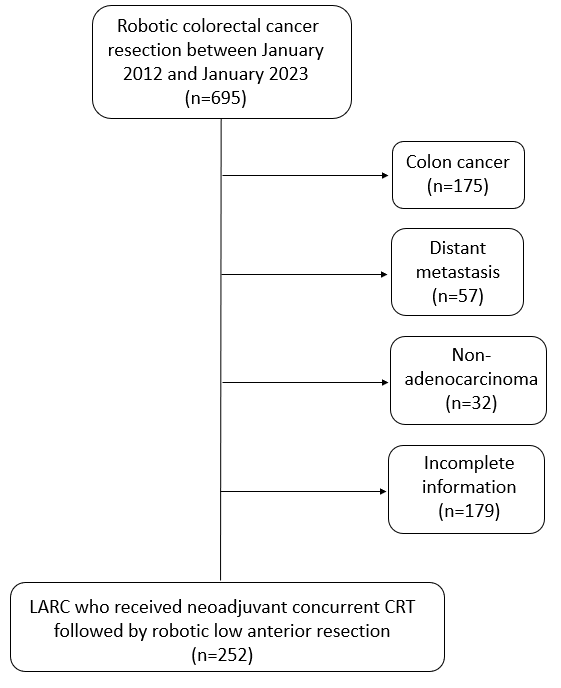


**Supplementary Figure S1.** Flowchart of patient selection. LARC: locally advanced rectal cancer; CRT: concurrent chemoradiotherapy.
